# A new species of *Aeneator* Finlay, 1926 (Mollusca, Gastropoda, Buccinidae) from northern Chile, with comments on the genus and a key to the Chilean species

**DOI:** 10.3897/zookeys.257.4446

**Published:** 2013-01-14

**Authors:** Juan Francisco Araya

**Affiliations:** 1 Universidad de Chile, Facultad de Ciencias, Las Palmeras 3425. Ñuñoa. Santiago. Chile

**Keywords:** New taxa, East Pacific, deep water

## Abstract

A new species of the genus *Aeneator* Finlay, 1926 is described from off the coast of Caldera (27°S), northern Chile. *Aeneator martae* sp. n. has a small, broad, stout, angulated shell with more prominent axial ribs and a more obviously keeled periphery than all previously named Chilean species. Comparisons are provided with all other South American named species of *Aeneator*.

## Introduction

The genus *Aeneator* Finlay, 1926 comprises a group of deep-water gastropods of moderate size, distributed in the South Pacific Ocean around New Zealand (Powell 1979, Beu 1979) and Chile (Rehder 1971, McLean and Andrade 1982, Fraussen and Sellanes 2008). Almost all the species have offshore distributions, and they are common on the sea floor (Dawson 1965, Powell 1979, Beu and Maxwell 1990). Their elongate fusiform shells have rounded whorls with a subsutural concavity, a lip with a broad shallow sinus below the suture, and a sculpture of strong axial ribs overridden by spiral cords (McLean and Andrade 1982).

In the southeastern Pacific the genus encompasses five extant species: *Aeneator castillai* McLean & Andrade, 1982, *Aeneator fontainei* (d’Orbigny, 1839), *Aeneator (Ellicea) loisae* Rehder, 1971, *Aeneator portentosus* Fraussen & Sellanes, 2008 and *Aeneator prognaviter* Fraussen & Sellanes, 2008. The distribution of these species ranges from Bahía Independencia (14°S), in the south of Peru to Canal Moraleda 45°22'S, southern Chile (Osorio et al. 2006). Their bathymetric range is from 10 m depth for *Aeneator fontainei* collected at Mejillones Bay, in the north of Chile (Guzmán et al. 1998, Laudien et al. 2007) to 800 m depth for *Aeneator portentosus*, collected off Iquique (Fraussen and Sellanes 2008). Most of the species have been recovered in the trawls of the local shrimp industry (McLean and Andrade 1982, Rehder 1971, Párraga 2012, Queirolo et al. 2011), and very little is known of their population biology, ecology and conservation status.

The present work describes a new species of *Aeneator* from northern Chile based on shell morphological features. Criteria were shell shape, number of primary spiral cords, development of secondary spirals, and axial sculpture. An identification key, based on shell characters, is given for all the extant Chilean *Aeneator* species.

## Material and methods

Material examined: *Aeneator martae* sp. n. types, Chile, Region of Atacama, Caldera, holotype MZUC 37890, paratype 1 MZUC 37891, paratype 2 MZUC 37892, paratype 3 MG 200105.

Examination was made of shell only specimens; all measurements were made with vernier callipers (± 0.1 mm). For the measure of length of aperture and angle of the spire, the methodology of Dépraz et al. (2009) and Chiu et al. (2002) was used.

Abbreviations: KF; Private collection of Mr Koen Fraussen, Aarschot, Belgium, MG: private collection of the author, section marine Gastropoda, MZUC; Museo de Zoología de la Universidad de Concepción, Concepción, Chile, RC Coll; private collection of Mr Ricardo Catalán, Servicio Nacional de Pesca, Chile.

## Results

### Systematics

Class: Gastropoda Cuvier, 1797

Order: Neogastropoda Wenz, 1938

Superfamily: Buccinoidea Rafinesque, 1815

Family: Buccinidae Rafinesque, 1815

**Genus: *Aeneator*** **Finlay, 1926:414**

**Type species.** *Verconella marshalli* Murdoch 1924 (by original designation), Pleistocene and recent, New Zealand.

#### Aeneator martae sp. n.

urn:lsid:zoobank.org:act:73AC9156-214E-4941-BFF6-0F94F8E17381

http://species-id.net/wiki/Aeneator_martae

Figs 1 –14 , 18 , Tables 1 , 2 , 3

##### Type material.

Holotype (MZUC 37890), 47.9 mm. Chile, off Caldera (27°04'S, 70°50'W), 550–600 m depth, live collected on shrimp trawl nets, January 2001, S. Castillo leg. Paratype 1 (MZUC 37891), length 44.0 mm. Paratype 2 (MZUC 37892), 41.7 mm, Paratype 3 (MG 200105), length 40.2 mm. All the paratypes with same locality as the holotype.

##### Distribution.

Known only from the type locality; Chile, Region de Atacama, Caldera (27°04'S, 70°50'W), 550–600 m depth.

##### Diagnosis.

A small species of *Aeneator*, height up to 47.9 mm, shell stout, inside of aperture pale orange, exterior sculptured by well-defined axial ribs, spiral cords, and a conspicuous stepped shoulder.

##### Description.

Shell small for genus (height up to 47.9 mm, Table 1), thick, solid, fusiform, chalky white to pale brownish, inside of aperture pale orange. Shape broad, angulate, length of aperture and canal more than half length of shell, width/height ratio 0.53 to 0.56, whorls convex apart from slightly concave sutural ramp, suture shallow but impressed. Spire angle 63° to 68°. Protoconch and upper teleoconch whorls missing, remaining whorls about 4.5, last 3 with sculpture intact with 7–9 primary spiral cords, interspaces each occupied by one narrow, well defined secondary cord. Last whorl with 16–18 spiral cords, more prominent at periphery of shell than elsewhere, forming a distinct keel. Spire whorls with 24–28 pronounced axial ribs, interspaces deep, each almost equal to a rib in width. Last whorl with 14–15 such ribs. Ribs more pronounced towards the anterior end of shell. Aperture ovate. Parietal and columellar area well-defined, glazed; outer lip thin, slightly crenulated, without lirae or teeth. Siphonal canal short, open, directed slightly to left. Operculum large, thin, dark brown, elongate, nucleus terminal, tip sharp.

**Table 1.** *Aeneator martae* sp. n. measurements of specimens. (%) means percentage compared to the total length of the shell.

	Maximum length (mm)	Maximum width (mm)	Length of aperture	Width/Length
Holotype	47.9	25.6	26.8 (56 %)	0.53
Paratype 1	44.0	23.6	27.9 (57 %)	0.54
Paratype 2	41.7	23.4	23.0 (57 %)	0.56
Paratype 3	40.2	22.1	22.6 (56 %)	0.55
Average	43.4	23.6	25.1 (56 %)	0.54

**Figures 1–5.**
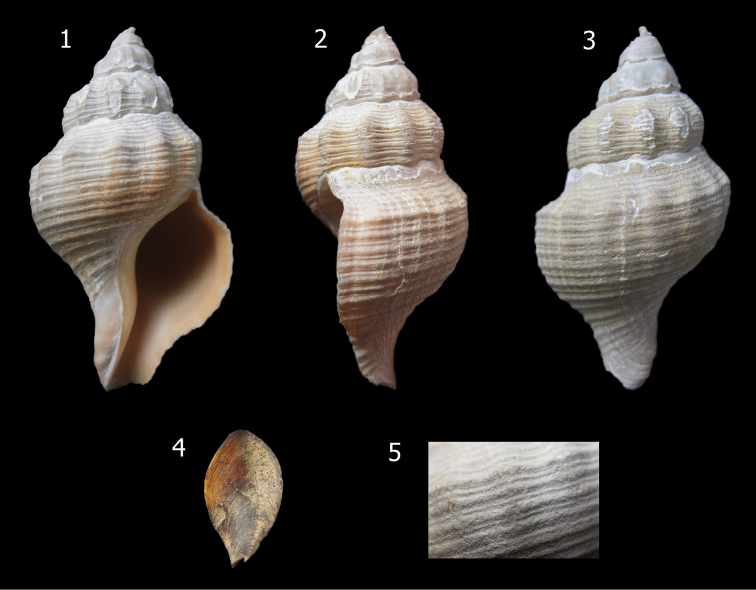
*Aeneator martae* sp. n. shell, Holotype 47.9 mm, Chile, Off Caldera, 27°04'S, 70°50'W. 550–600 m. MZUC 37890.

**Figures 6–14.**
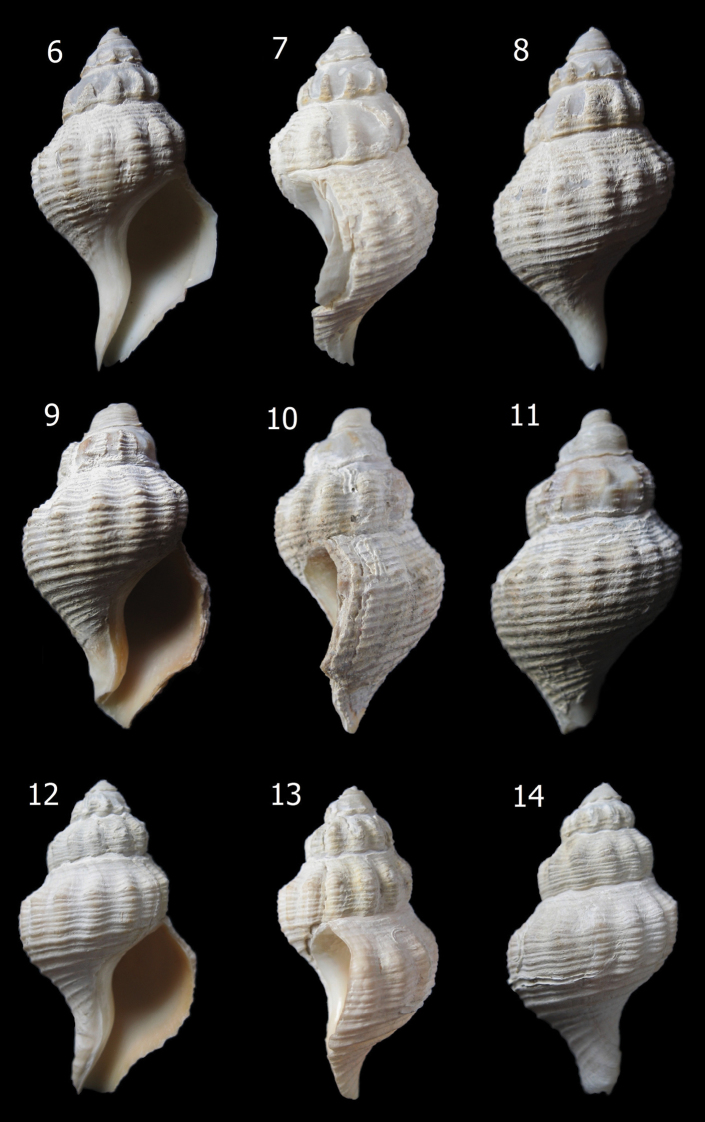
*Aeneator martae* sp. n. shells. **6–8** Paratype 1 (MZUC 37891), 44.0 mm height **9–11** Paratype 2 (MZUC 37892), 41.7 mm height **12–14** Paratype 3 (MG 200105), 40.2 mm height.

**Figures 15–20.**
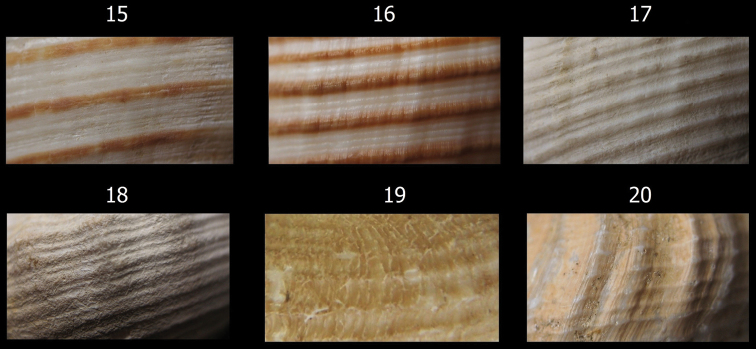
Details of shell sculpture of Chilean*Aeneator* species. **15** *Aeneator castillai* (RC Coll.), 85.7 mm **16** *Aeneator fontainei* (RC Coll.), 48.0 mm **17** *Aeneator loisae* (MG 200003), 78 mm **18** *Aeneator martae* sp. n. paraype 3 (MG 200105), 40.2 mm **19** *Aeneator portentosus*, Paratype KF-0338, 45.5 mm **20** *Aeneator prognaviter* (MG 200124), 33,0 mm.

##### Etymology.

Named in honour of Mrs Marta Araya,Caldera, Chile, who presented the specimens to the author.

##### Remarks.

In Chile the genus *Aeneator* encompasses five extant species: *Aeneator castillai*, found from Coquimbo (29°55'S) to Punta Peñablanca (33°22'S) in 200–450 m (McLean and Andrade 1982), *Aeneator fontainei*, the most common species, with records from Bahía Independencia (14°S) in the south of Peru (McLean and Andrade 1982) to Estero Elefantes, 46°05'S (Osorio et al. 2006) and with a bathymetric range of 10 m near Mejillones (Guzmán et al. 1998, Laudien et al. 2007) to 421 m for a specimen collected off Coquimbo (Figs 27–30), *Aeneator (Ellicea) loisae*, distributed from Caldera (27°04'S), for material examined in this work (Figs 27–30), to Canal Moraleda (45°22'S), in the fjords area (Osorio et al. 2006) with a bathymetric range of 200 m (McLean & Andrade 1982) to 465 m, *Aeneator portentosus* reported only form the original locality off Iquique (21°19'S) in 605 m and off Coquimbo at 800 m and *Aeneator prognaviter*, distributed off Antofagasta (22°51'S) in 318 m (Fraussen and Sellanes 2008) and in 748 m off Iquique for material examined in this work (Fig. 37). Data on the localities of Chilean species of *Aeneator* is provided in Table 3.

**Table 2.** Synthesis of characters of the Chilean species of *Aeneator* Finlay, 1926 based on Rehder (1971), McLean and Andrade (1982), Fraussen and Sellanes (2008) and material examined in this work.

	*Aeneator castillai*	*Aeneator fontainei*	*Aeneator loisae*	*Aeneator portentosus*	*Aeneator prognaviter*	*Aeneator martae* sp. n.
Length	85.7	85.8	104	45.5	32.2	47.9
Width / Length	0.50–0.51	0.48–0.54	0.43–0.48	0.49–0.59	0.55 – 0.60	0.54–0.56
Aperture length/ total length	0.55–0.63	0.55–0.56	0.54 – 0.56	0.43–0.52	0.49 – 0.53	0.55–0.56
Spire angle	50°	51°–57°	44°–46°	44°–51°	60°	63°–68°
Axial ribs on last whorl	16, absent in subsutural area	12–15	Faint, absent	Faint, absent	22, bent	14–15, straight
Spiral cords in last whorl	12 – 15 brown primary, 1–3 secondary in each interspace	12–15 brown primary, 3–5 secondary in each interspace	9–10 primary, many secondary	20	20–24	16–18 primary, 7–9 secondary
Siphonal canal	short, twisted	medium to long, straight	long	short, broad, slightly bent	short, broad	short, slightly curved to left
Aperture	ovate	ovate	elongate ovate	round	oval	oval
Shell color	brown	white - yellowish	white	snow white	snow white	white, pale brownish
Distribution	29°55'S to 39.1°S	14°13'S to 46°S	27°04'S to 53.7°S	21.19°S and 29.95°S	21°19'S and 22°51'S	27°04'S

**Table 3.** Table of localities of Chilean species of *Aeneator* Finlay, 1926 based on Rehder (1971), McLean and Andrade (1982), Fraussen and Sellanes (2008), and material examined in this work.

Species	Latitude	Longitude	Depth (m)
*Aeneator castillai*	29°55'S to 33°22'S	71°53'W to 71°20'W	200–450
*Aeneator fontainei*	14°14'S to 46°05'S	76°11'W to 73°41'W	10–421
*Aeneator loisae*	27°04'S to 45°22'S	73°21'W to 70°50'W	200–465
*Aeneator martae*	27°04'S	70°50'W	550–600
*Aeneator portentosus*	21°19'S to 29°55'S	71°20'W to 70°09'W	800
*Aeneator prognaviter*	21°19'S to 22°51'S	70°24'W to 70°09'W	600–748

**Figures 21–26.**
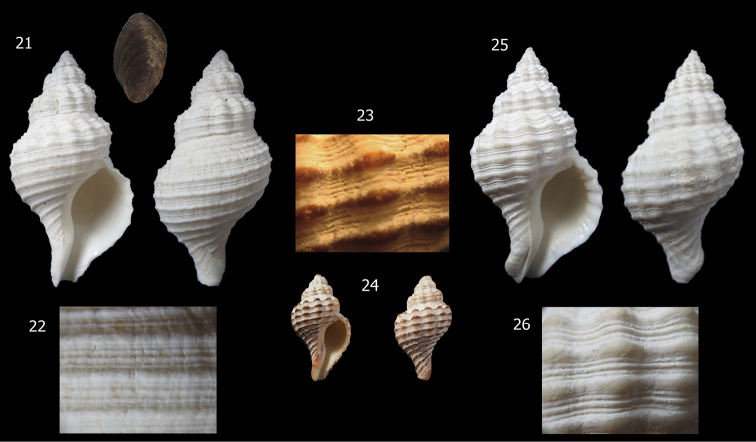
*Aeneator fontainei* varieties and details of shell sculpture. **21–22** Off Coquimbo, Chile, trawled 421 m (RC Coll), 58 mm **23–24** Washed ashore, Calderilla beach, Caldera, Chile (MG 200011), 28.5 mm **25–26** Dredged 20 m depth off Loreto beach, Caldera, Chile (MG 200012), 52.8 mm.

**Figures 27–32.**
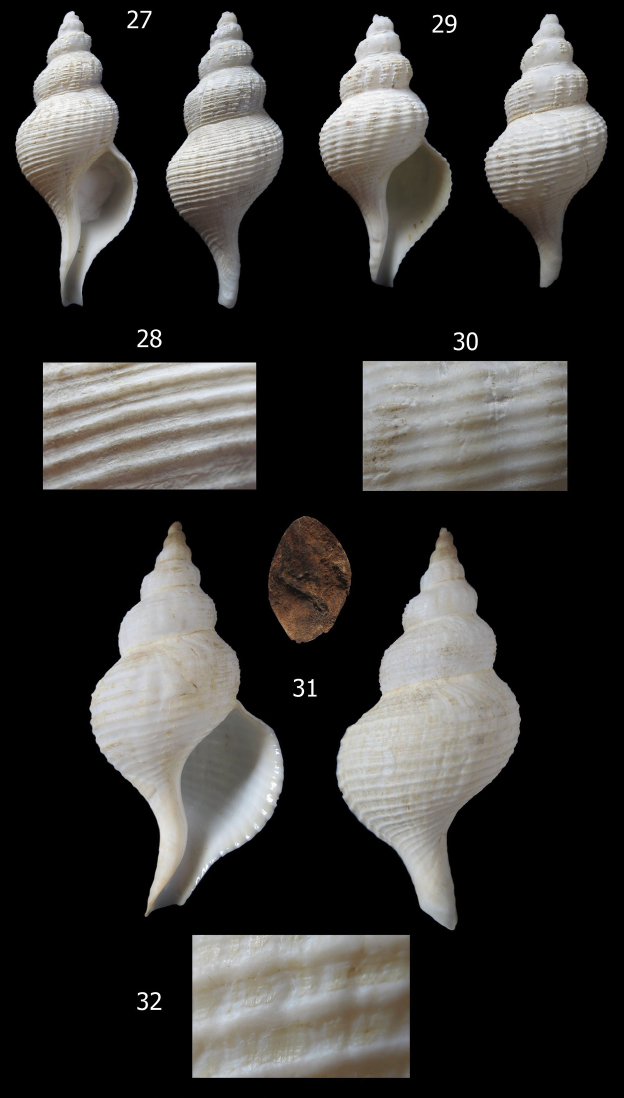
*Aeneator loisae* varieties and details of shell sculpture **27–28** Off Caldera, Chile, 450–500 m depth (MG 200003), 78 mm **29–30** Off Caldera, Chile, 420 m depth (MG 200007) 71.9 mm **31–32** Off Coquimbo, Chile, 400 m depth (RC Coll.), 104 mm

In size, the shell of *Aeneator martae* sp. n. is similar to *Aeneator prognaviter* (Figs 20, 37, 38) and *Aeneator portentosus* (Figs 35, 36). However, the former of these two can be clearly differentiated from the new species by its wider and shorter siphonal canal, less numerous and more curved axial ribs and a thinner, snow white shell (Fraussen and Sellanes 2008). From *Aeneator portentosus* the new species differs by having a much wider, thicker shell with a shorter spire, a more elongate aperture, dominant axial sculpture and less rounded whorls. Moreover *Aeneator portentosus* exhibit a very distinctively sculptured periostracum (Fig. 19), with low axial ridges, very different from all the other Chilean *Aeneator* species. A periostracum is absent in the examined specimens of *Aeneator martae* sp. n.

**Figures 33–38.**
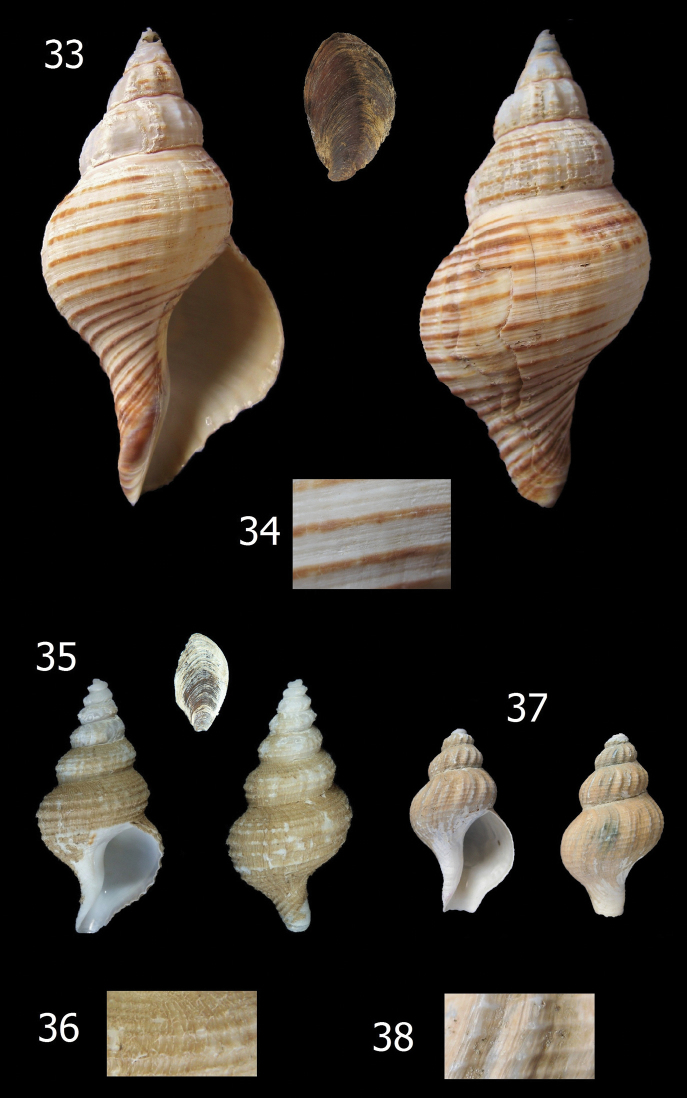
*Aeneator* species and details of shell sculpture. **33–34** *Aeneator castillai*, off Coquimbo, Chile, 380 m depth (RC Coll.), 85.7 mm **35–36** *Aeneator portentosus*, Paratype KF-0338, 45.5 mm **37–38** *Aeneator prognaviter*, off Iquique, Chile, 748 m depth (MG 200124), 33,0 mm.

*Aeneator castillai* (Figs 33, 34), and *Aeneator fontainei* (Figs 21–26) differ markedly from the new species by their much larger shells, reaching up to 85.8 mm, more fusiform shells, with a much less stepped or indistinct shoulder, lower and fewer axial ribs, brown primary spiral cords (Figs 15, 16) and lip lirated within. The spiral sculpture is quite different; *Aeneator fontainei* has 13 to 16 dark brown major cords, with interspaces filled with five secondary cords separated by fine grooves or by secondary and tertiary cords. *Aeneator castillai* has brown primary cords with 3 to 5 fine secondary cords filling the interspaces and exhibits a longer, twisted, siphonal canal. In contrast *Aeneator martae* sp. n. lacks any brown coloration, shows a sculpture of alternated single major and minor spiral cords defined mostly in the posterior part of the whorls, and has a conspicuous stepped shoulder, forming a keel at the periphery.

*Aeneator loisae* (Figs 27–32) differs from the new species in having a larger, up to 104 mm, white to snow white shell (different from the white to light brown shell of *Aeneator martae* sp. n.), more inflated last whorl, with a much longer siphonal canal, a higher number of primary and secondary spiral cords, more prominent spiral sculpture, and fewer, more tenuous, axial ribs.

The new species is tentatively assigned, given the generic uncertainties within the Chilean species, to the genus *Aeneator* Finlay 1926, typified by the species *Aeneator marshalli marshalli* (Murdoch, 1924) recorded from Castlecliff (as fossils) and, as a recent species (= *Aeneator marshalli separabilis* Dell, 1956), from Wanganui and Ohope beach, Whakatane, New Zealand. Similar to the type species, *Aeneator martae* sp. n. has a fusiform shell with moderately tall spire, shallow sinus in outer lip and a spiral sculpture of cords crossed by axial costae (Beu and Maxwell 1990). The new species differs from *Aeneator marshalli* in its smaller shell, shorter anterior canal, the absence of nodules along the columellar lip, less inflated whorls and by the presence of a distinct keel at the periphery. From the genus *Austrofusus* Kobelt, 1879, with the type species *Austrofusus glans* (Röding, 1798), the new species differs in the smaller size, its thicker shell, more prominent sculpture, the more prominent ridges over the periphery, and the pale orange colour of the aperture, which is white in *Aeneator glans* (Beu & Marshall 2010). Comparative characters in the Chilean species of *Aeneator* are compared in table 2.

In a recent revision of the fossil fauna of Mejillones, north of Chile (Nielsen 2012), the species *Aeneator loisae* was synonymized with the fossil species *Fusus steinmanni* Möricke, 1896 into *Austrofusus*. However, this was based partly on the incorrect conclusion by Beu and Marshall (2010) that *Aeneator fontainei* is the type species of *Austrofusus*; this was later corrected by Beu and Marshall (2011). On morphological grounds, the author concurs with McLean and Andrade (1982) and considers that *Aeneator (Ellicea) loisae* does belong to the genus *Aeneator* and the sub-genus *Ellicea* Finlay in Marwick, 1928. However the generic placement of the species *Aeneator fontainei*, *Aeneator castillai*, and possibly the new species described here, should be further investigated or even be ascribed to a new genus.

Further study of radular characters, comparative anatomy and DNA will improve the taxonomic placement of the Chilean species. Fossil studies would also give a general insight into the development of the genus and their relationships with the South Pacific related fauna, especially those from New Zealand and adjacent waters.

##### Comparative material examined:

*Aeneator castillai*, Chile, Region of Coquimbo, Coquimbo, 2 specimens RC Coll. *Aeneator fontainei*, Chile, Region of Atacama, Caldera, 3 specimens MG 200011–200013, 5 specimens RC Coll. *Aeneator loisae*, Chile, Region of Atacama, Chile, 4 specimens MG 200003–200006, 1 specimen RC Coll, *Aeneator prognaviter*, 2 specimens MG 200124–200125, *Aeneator portentosus*, 1 specimen (examined from images), KF-0338.

### Key for the identification of fully-grown Chilean species of *Aeneator* based on shell characters

**Table:** 

1	Aperture ovate-elongate	2
–	Aperture rounded, shell pagodoid, periostracum sculptured	*Aeneator portentosus* Fraussen & Sellanes, 2008
2(1)	Siphonal canal short	3
–	Siphonal canal long, outer lip reflexed, shell elongated	*Aeneator loisae* Rehder, 1971
3(2)	Spiral cords brown	5
–	Spiral cords white, axial ribs thick, shell length up to 49 mm	4
4(3)	Siphonal canal broad, axial ribs strongly curved	*Aeneator prognaviter* Fraussen & Sellanes, 2008
–	Shell with a distinct keel, aperture almost subquadrate	*Aeneator martae* sp. n.
5(3)	Axial ribs on subsutural area	*Aeneator fontanei* (d’Orbigny, 1841)
–	Sculpture absent on subsutural area, siphonal canal twisted	*Aeneator castillai* MacLean & Andrade, 1982

## Supplementary Material

XML Treatment for Aeneator martae
